# Limited discectomy versus aggressive discectomy by spinal endoscopy with the transforaminal approach for lumbar disc herniation: a retrospective study

**DOI:** 10.1186/s12891-024-07498-8

**Published:** 2024-05-28

**Authors:** Sulaiman Reheman, XiangYu Meng, Tuerhongjiang Abudurexiti, Abuduwupuer Haibier, Weibin Sheng

**Affiliations:** 1 First Affiliated Hospital of Xinjiang Medical University, No.137 Liyushan Road, Urumqi, Xinjiang Uygur Autonomous Region People’s Republic of China; 2 Sixth Affiliated Hospital of Xinjiang Medical University, Xinjiang Uygur Autonomous Region, Urumqi, People’s Republic of China

**Keywords:** Lumbar disc herniation, limited discectomy, aggressive discectomy, percutaneous endoscopic transforaminal discectomy

## Abstract

**Objective:**

To compare the clinical and radiological outcomes of limited discectomy (LD) and aggressive discectomy (AD) performed via spinal endoscopy using the transforaminal approach in patients with lumbar disc herniation(LDH)

**Methods:**

We conducted a retrospective review of patients who underwent percutaneous endoscopic transforaminal discectomy (PETD) at the L4-L5 lumbar spine segments in our department from January 2017 to December 2020. The follow-up period extended to 24 months postoperatively. Patients were categorized into the LD and AD groups based on the extent of intraoperative disc removal. We retrospectively collected and analyzed clinical and radiological data.

**Results:**

The study followed 65 patients, with 36 in the LD group and 29 in the AD group. No statistically significant differences were noted in recurrence rates, the excellent and good Macnab rates, preoperative Disc Height Index (DHI), and preoperative Modic changes between the groups (*P* >0.05). However, significant differences were observed in operation duration, postoperative DHI and postoperative Modic change (*P*<0.05). No significant differences in Visual Analog Scale (VAS) and Oswestry Disability Index (ODI) scores were detected between the groups preoperatively, or one and two years postoperatively (*P*>0.05). Nevertheless, notable differences in VAS and ODI scores were present one month postoperatively (*P*<0.05).

**Conclusion:**

As a conventional surgical method for treating LDH, PETD can achieve satisfactory clinical results in both LD and AD, with no significant variance in recurrence rates. However, AD is associated with longer operation times, and greater postoperative reductions in DHI and greater postoperative Modic changes compared to LD.

## Introduction

Lumbar disc herniation (LDH) is a prevalent degenerative condition of the spine, manifesting as back and leg pain. Lumbar discectomy stands out as the predominant surgical intervention for LDH. While the herniated fragment is widely recognized as the culprit, conventional open surgery seeks neural decompression by removing an extensive portion of disc tissue—termed aggressive discectomy (AD)—to diminish the risk of postoperative recurrence. This principle has seldom been scrutinized despite the paucity of empirical support.

Williams [1, 2] was the first to introduce the concept of limited discectomy (LD), applicable primarily to a small subgroup of patients with a free fragment compressing the nerve root (≤ 10% of the overall population with disc herniations). He reported a recurrence rate of 4–9% and a clinical success rate of 90%. Subsequent studies by other researchers confirmed these results [3, 4], as summarized by Wenger et al. [5]. However, researchers such as Park JS et al. [6], Balderston et al. [7], and Kahanovitz et al. [8] found no significant difference in recurrence rates, whereas Rogers [9] and Carragee et al. [10] demonstrated a higher incidence of recurrent disc herniation with LD. In contrast, Faulhauer [2] noted a lower recurrence rate with LD compared to AD. It is important to acknowledge that these studies were generally underpowered to effectively evaluate differences in recurrent disc herniation, and the impact of short-term follow-up on these findings remains uncertain.

The systematic review by McGirt et al. [11] revealed a higher incidence of long-term recurrent back and leg pain after AD, yet a higher incidence of recurrent disc herniation following LD, drawing upon studies related to open surgery, known for poor long-term outcomes and extended recovery periods [12].

With advancements in minimally invasive surgery, Soliman et al. [8] observed an 11.1% recurrence rate in patients undergoing minimally invasive decompression after eight years, noting a significant long-term benefit over open surgery.

Currently, PETD is regarded as a conventional surgical technique for LDH. Distinct from other methods, it causes minimal or no damage to the facet joints, which are essential for spinal stability. To date, no studies have compared the clinical and radiographical outcomes of LD and AD using percutaneous endoscopy for lumbar disc herniation.

In our research, LD is characterized as identifying and removing only the herniated disc fragment from the weakened or ruptured area of the intervertebral disc annulus, without intervening in the intervertebral disc itself. Conversely, AD involves deliberately breaching the annulus fibrosus, excising the herniated disc material, removing as much intervertebral tissue as feasible, and scraping the end plates.

The objective of our study is to retrospectively assess LD versus AD through percutaneous endoscopy, focusing on clinical and radiological outcomes and, notably, on the recurrence rate.

## Objectives and methods

### Design

Retrospective comparative study.

### Time and location

This study was conducted at the Department of Spine Surgery, Sixth Affiliated Hospital of Xinjiang Medical University, from January 2017 to December 2020.

### Inclusion criteria

(i) Presentation of radicular leg pain
(ii) L4–L5 lumbar disc herniation confirmed by CT and MRI
(iii) Informed consent provided by all participants, approved by the Ethics Committee of the Sixth Affiliated Hospital of Xinjiang Medical University
(iv) Patients with comprehensive clinical and radiographic data
(v) Non-responsiveness to conservative therapy for at least 3 months before surgery

### Exclusion criteria

(i) Patients presenting with lumbar instability
(ii) Bone infection
(iii) Systemic diseases
(iv) History of lumbar surgery or trauma
(v) Body mass index exceeding 35 kg/m².

### Surgical method

#### Preoperative preparation

Comprehensive diagnostic evaluations were conducted preoperatively, including x-ray, CT scan, and MRI, alongside routine hematological assessments, and liver and kidney function tests.

#### Surgery method

Patients were positioned prone with knees and hips flexed to indirectly enlarge the intervertebral foramen. A C-arm fluoroscope was positioned near the head, perpendicular to the patients, ensuring precise frontal and lateral X-ray images. Using a fluoroscopic-guided postero-lateral, transforaminal approach, a puncture needle was positioned in the intervertebral foramen, anterior to the facet joint. Subsequently, a working channel endoscope was inserted for direct visualization and irrigation. The ventral part of the L5 upper facet joint was resected using a trephine or bone drill to facilitate a safe transforaminal approach. The ligamentum flavum was then dissected or removed, enabling endoscopic access to the spinal canal. Discectomy was performed as needed, followed by decompression until fluid pulsations resumed. Bleeding vessels and the injured annulus fibrosus were coagulated with a radiofrequency bipolar coagulator. The procedure concluded with a visual inspection of the nerve root while gradually withdrawing the working channel and endoscope.

#### Postoperative management

Post-surgery, patients were required to remain recumbent for 6–8 h. During mobilization, they wore a thoracolumbar support belt to aid walking. An MRI was conducted 3 to 4 days postoperatively.

### General information

This study was conducted at the Department of Spine Surgery, Sixth Affiliated Hospital of Xinjiang Medical University, from January 2017 to December 2020.

Patients were categorized into two groups by third-party observers based on the surgical method—LD or AD—as determined from endoscopic surgery videos. All procedures were performed by an attending surgeon, with a follow-up duration of 24 months post-surgery. Demographic and perioperative data, including gender, age, BMI, comorbidities (e.g., hypertension, diabetes), intraoperative blood loss, and operation duration, were recorded.

### Observed indicators

#### Primary outcome measures

The following parameters were meticulously evaluated at designated intervals:

- Visual analogue scale (VAS) scores for back pain at preoperative, 1 week, 1, 12, 24 months postoperative intervals,
- Oswestry Disability Index (ODI) scores before surgery and at 1 week, 1, 12, 24 months postoperative intervals,
- Modified MacNab score, assessed at the final follow-up,
- Reherniation rates were recorded.

The VAS is a diagnostic tool that measures pain intensity on a scale from 0 (no pain) to 10 (maximum pain). The ODI evaluates functional improvement in various domains, including pain intensity, lifting, walking, sleeping, and social interaction. An ODI score of 0% indicates minimal disability, while 100% denotes severe disability, possibly necessitating bed rest or indicating the presence of exaggerated symptoms.

#### Secondary outcome measures

**Fig. 1:**
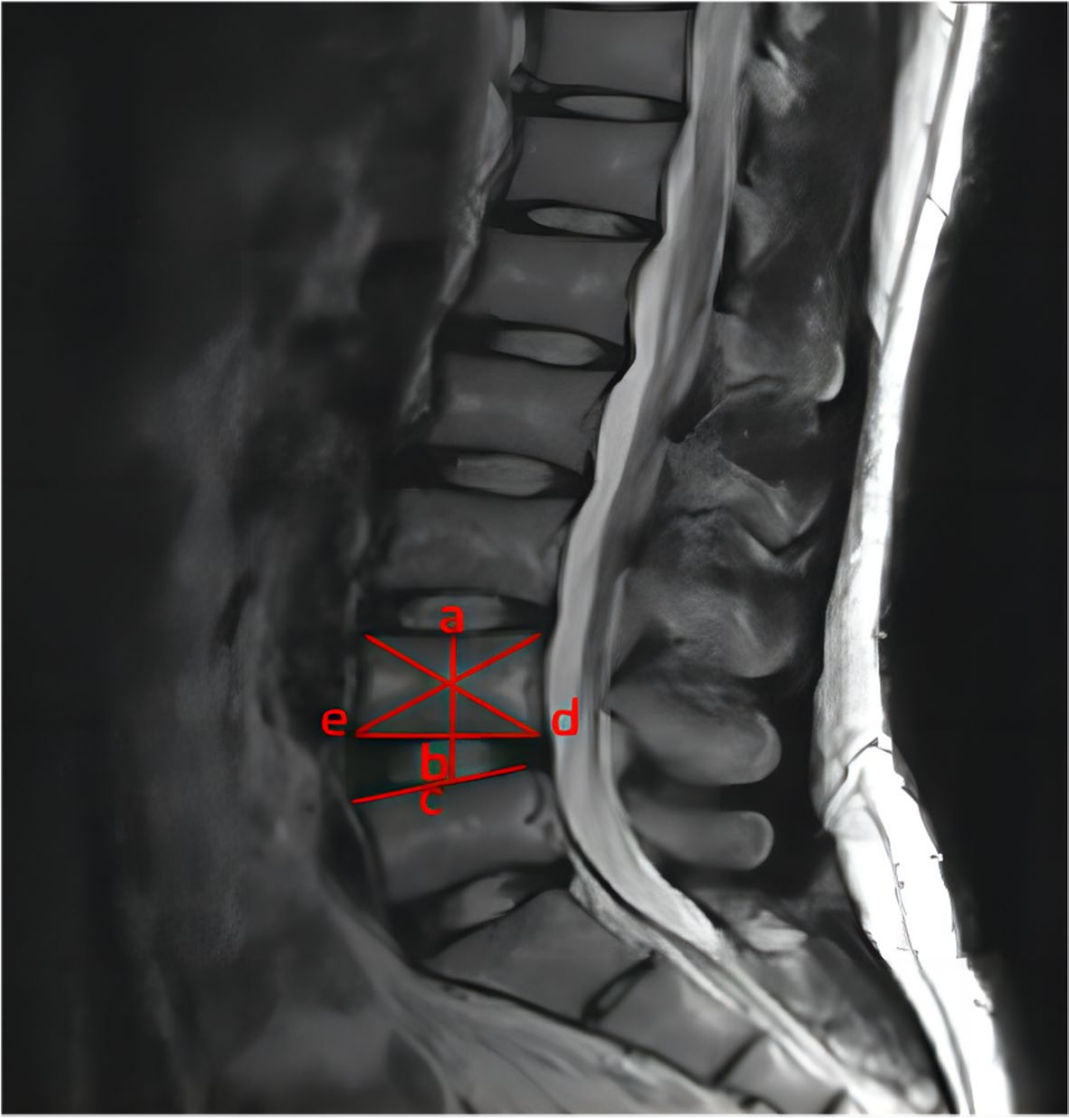
Disc Height Index (DHI). DHI=bc/ab (The line ac is perpendicular to line de; DHI is the ratio of line bc to line ab)

Disc Height Index (DHI) (Fig. 1) and Modic changes were assessed preoperatively and postoperatively at 1 and 2 years.

Recurrent LDH was defined as the reappearance of symptoms and signs post-initial surgery, with subsequent MRI confirmation of disc herniation. Patients with persistent symptoms post-surgery were excluded. An MRI was conducted 3 to 4 days post-surgery to eliminate cases with residual disc material. Recurrence implied the patient exhibited nearly identical pre-surgery symptoms. At this juncture, an MRI was revisited, and findings were compared with baseline MRI images.

### Statistical methods

 All statistical analyses were executed using SPSS 22.0. Numerical continuous variables are expressed as mean ± standard deviation. Data were subjected to the t-test, chi-square, or univariate logistic regression analysis, as applicable. A *p*-value < 0.05 was deemed statistically significant.

## Results

### Participant analysis

The study comprised 65 patients, with 36 in the LD group (20 males, 16 females; average age 52.20 ± 16.13 years) and 29 in the AD group (16 males, 13 females; average age 51.34 ± 14.75 years). All participants were fully included in the outcome assessment without any data exclusion.

### Experimental flow chart

**Fig. 2:**
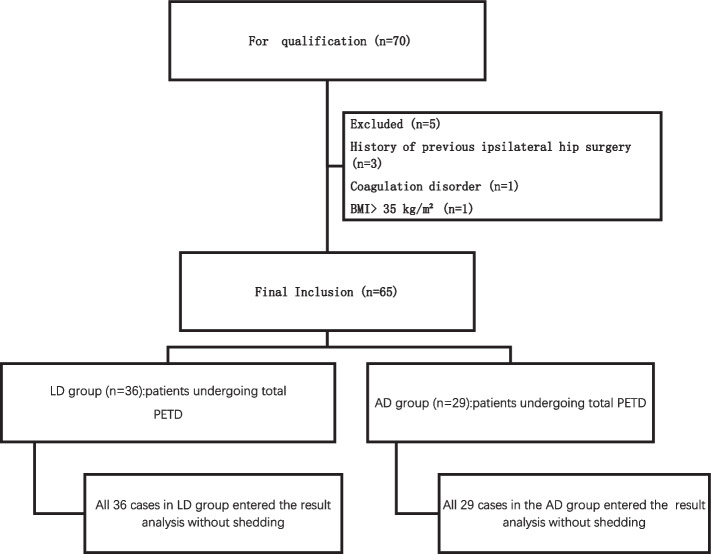
Flow chart of test grouping

The flow chart delineating the two groups is depicted in Fig. 2.

### Comparison of preoperative data between the two groups

Upon rigorous statistical scrutiny, no significant differences were noted in age, gender, BMI, comorbidities, and intraoperative blood loss between the two groups (*P* > 0.05). However, a significant difference was observed in operation time (*P* < 0.05) (Table 1).

**Table 1:** Comparison of general data between the two groups

	LD (*n* = 36)	AD (*n* = 29)	*P* value
Age (year)	52.20 ± 16.13	51.34 ± 14.75	0.817
Sex (M/F)	20/16	16/13	0.916
BMI (kg/m^2^)	25.12 ± 2.34	24.89 ± 2.45	0.689
Comorbidities			
hypertension	11(30.56%)	9(31.03%)	0.967
diabetes	4(11.11%)	3(10.35%)	0.921
Operation time (min)	61.23 ± 4.28	98.15 ± 6.26	0.029
Intraoperative blood loss (ml)	42.93 ± 3.19	48.62 ± 3.74	0.140

### Comparison of preoperative and postoperative VAS, ODI, and Macnab scores between the two groups

No significant difference was found in preoperative VAS and ODI scores between the groups (*P* > 0.05). However, 1 month post-surgery, the LD group exhibited greater improvement in VAS and ODI scores than the AD group, with the difference being statistically significant (*P* < 0.05). Patients reported continued pain relief during telephone follow-ups. VAS and ODI scores significantly improved at 1, 12, and 24 months post-surgery, with no significant differences between the groups (*P* > 0.05). At 12 and 24 months post-surgery, DHI decreased in both groups, with statistically significant differences observed between them (*P* < 0.05). No significant differences in Modic changes were noted between the LD and AD groups preoperatively and at 12 months post-surgery (*P* > 0.05). However, a significant difference in Modic changes was identified at 24 months post-surgery. The total recurrence rate was 9.2% (7 cases), occurring within 12 months post-surgery, with 4 cases (11.11%) in the LD group and 3 cases (10.3%) in the AD group. The excellent and good rate of the MacNab score was 91.7% in the LD group and 89.7% in the AD group. At the final follow-up, no significant difference was observed in reherniation rates or Modified MacNab scores between the groups (*P* > 0.05) (Table 2).

**Table 2:** Comparison of the clinical and imagiological outcome between the two groups

Group	LD	AD	*P*
Preoperative VAS	6.25 ± 0.95	6.09 ± 0.64	0.410
Postoperative VAS			
One month	1.80 ± 0.97	2.41 ± 0.88	0.007
One year	1.48 ± 1.56	1.72 ± 1.40	0.497
Two years	1.05 ± 0.97	1.26 ± 1.19	0.586
Preoperative ODI	67.9% ± 9.0%	65.3%±8.1%	0.210
Postoperative ODI			
One month	26.1%±7.6%	30.4%±5.5%	0.009
One year	11.2%±12.3%	11.3%±9.2%	0.958
Two years	9.47%±8.28%	10.56%±10.77%	0.792
Preoperative DHI	11.10 ± 2.14	11.06 ± 3.07	0.930
Postoperative DHI			
One year	10.80 ± 2.01	9.76 ± 1.69	0.046
Two years	10.62 ± 1.81	9.47 ± 1.45	0.014
Modic changes(yes/no)			
Preoperative	7/29	6/23	0.901
Postoperative	7/29	6/23	0.901
One year	7/29	11/18	0.098
Two years	7/29	13/16	0.028
Modified MacNab score (%)			
final follow-up	91.7%	89.7%	0.921
Recurrence rate			
One year	11.1%	10.4%	0.921
Two years	11.1%	10.4%	0.921

### Typical cases: see Figs. 3 and 4

**Fig. 3:**
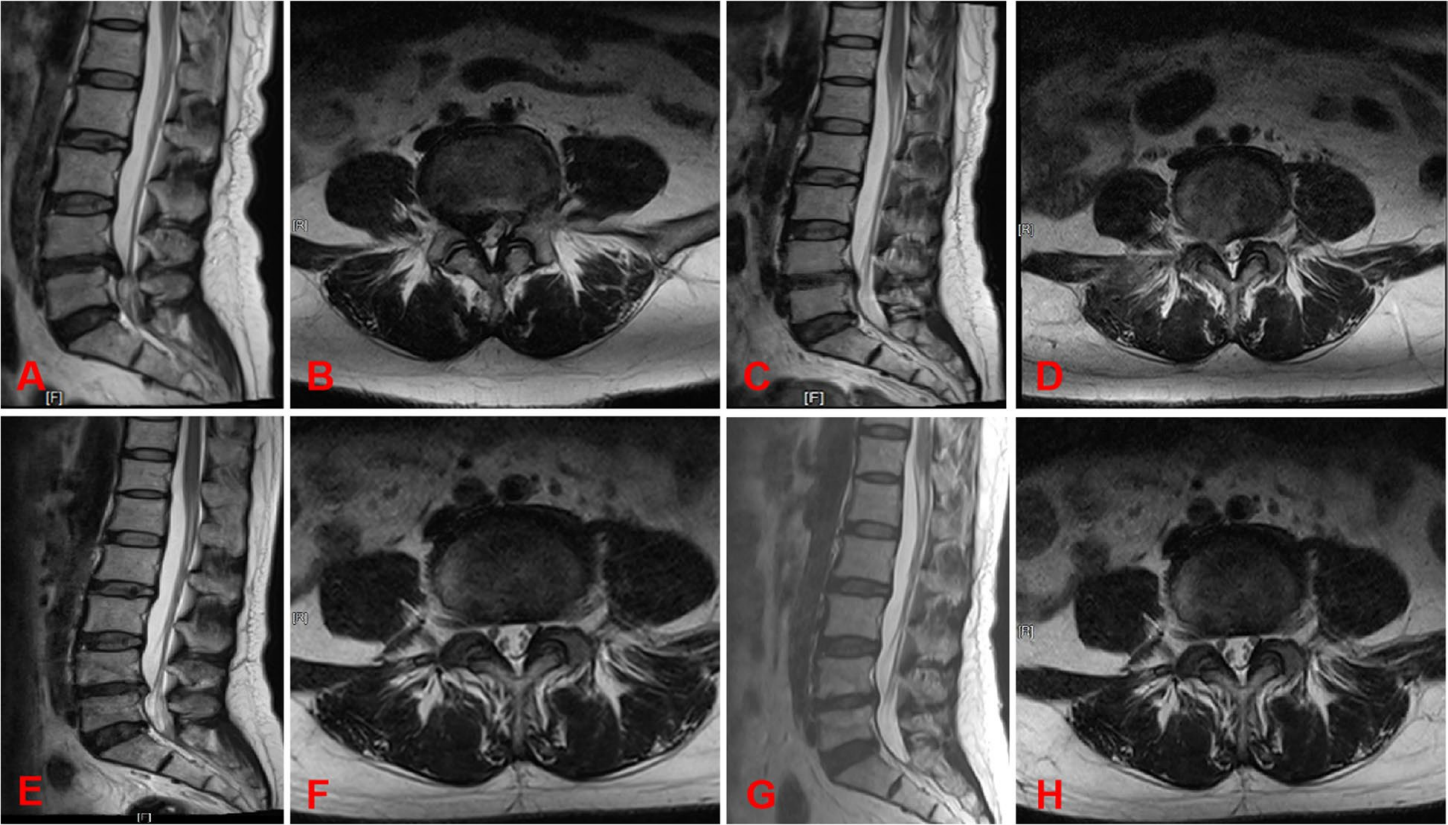
Aggressive discectomy. **A**-**B** Preoperative sagittal MRI and cross-sectional MRI; **C**-**D** sagittal MRI and cross-sectional MRI 3 days after surgery; **E**-**F** sagittal MRI and cross-sectional MRI 1 year after surgery; **G**-**H** sagittal MRI and cross-sectional MRI 2 years after surgery

**Fig. 4:**
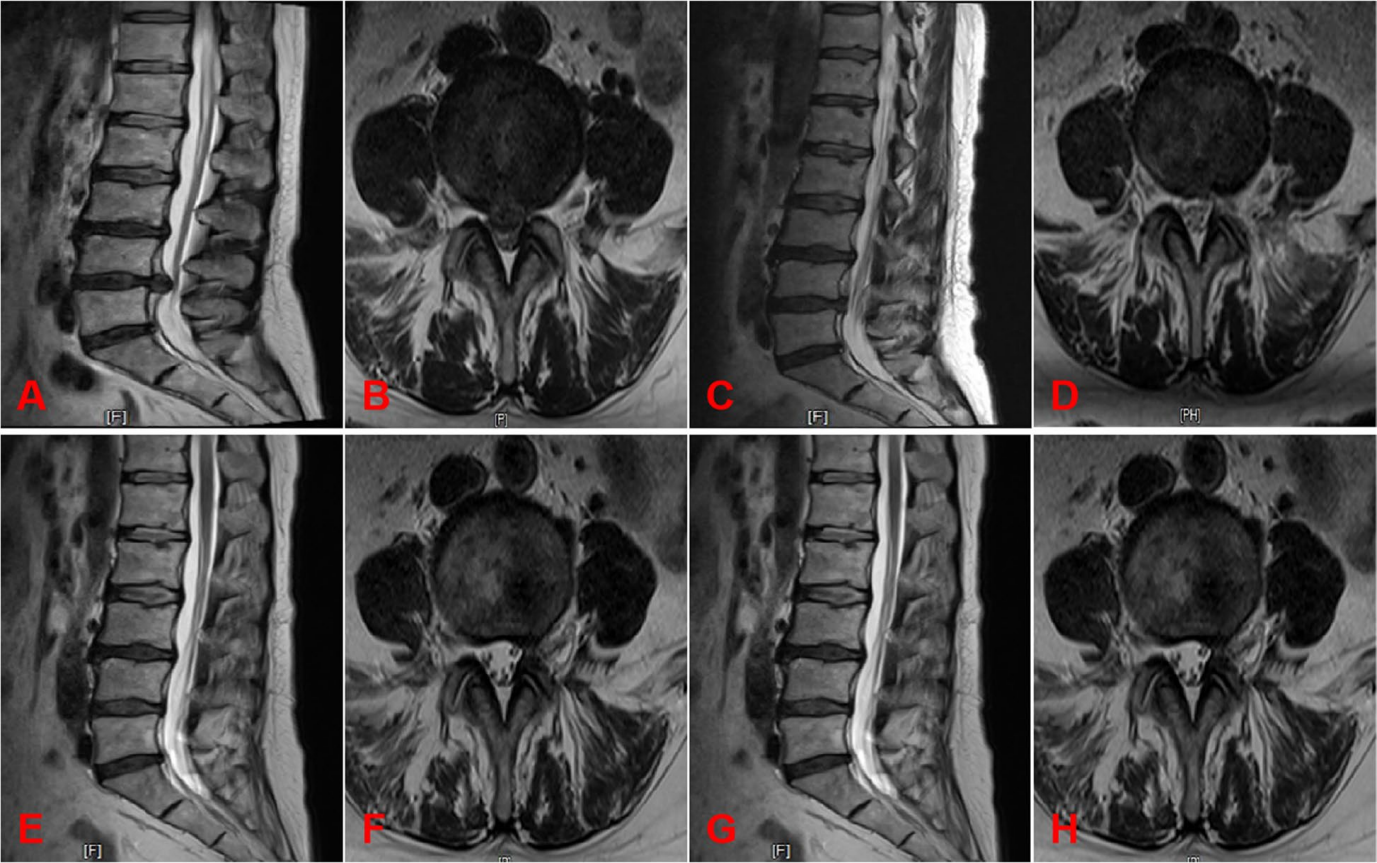
Limited Discectomy. **A-B** Preoperative sagittal MRI and cross-sectional MRI; **C**-**D** sagittal MRI and cross-sectional MRI 3 days after surgery; **E**-**F** sagittal MRI and cross-sectional MRI 1 year after surgery; **G**-**H** sagittal MRI and cross-sectional MRI 2 years after surgery

## Discussion

There is no standardized criterion for determining the completion of decompression and discectomy in lumbar disc herniation treatment. Historically, conventional surgery aimed to remove as much intervertebral disc tissue as feasible to reduce recurrence risk, guided by the limited intraoperative visibility characteristic of open procedures.

The advent of minimally invasive spinal technologies, particularly endoscopic spinal surgery, has provided surgeons with an enhanced endoscopic view, revealing structures unseen in open surgery, such as compromised or ruptured intervertebral disc annuli and herniated fragment configurations. PETD is considered a conventional surgical method for the treatment of LDH. Unlike traditional surgical approach, there is no damage to the facet joints which is crucial to the stability of the spine. Observations during endoscopic surgery revealed that herniated fragments, considered normal disc tissue crucial for the stability of the anterior and middle spinal columns, had detached from the main disc body. This detachment occurred irrespective of the herniation type (contained or extruded), indicating that the fragments were now isolated from the intervertebral disc tissue. This suggests a potential equilibration within the intervertebral disc post-herniation. In revision discectomies, we noted that reherniation could stem from the disc’s separation from adjacent structures, a process that might be unavoidable in some instances. These insights prompted us to delve into a comparative study of LD versus AD, offering a new perspective in our field [13].

In our study, we observed no statistically significant difference in the postoperative recurrence rates between LD and AD, which may be attributed to the minimally invasive nature of PETD. In PETD, the posterior columns of the spine are almost unaffected, thus enhancing spinal stability compared to open surgery. However, we identified a statistically significant difference in postoperative Modic changes between LD and AD. Additionally, a decrease in DHI was noted, more markedly in AD than in LD, with this difference being statistically significant.

LD specifically targets the removal of free fragments from the intervertebral disc tissue, avoiding unnecessary intervention in the intervertebral disc itself. This approach minimizes damage to the annulus, preserves the structural integrity of the anterior and middle columns, and potentially reduces postoperative degeneration. Such preservation may prevent or diminish the risks associated with degeneration, potentially lowering the incidence of failed back syndrome. Furthermore, it has been demonstrated that limited discectomy results in superior postoperative imaging and clinical outcomes [14].

AD entails the extensive resection of intervertebral tissue and endplate curettage. Such extensive removal of anterior column tissue during discectomy may compromise the structural integrity of the lumbar vertebrae and potentially hasten intervertebral disc degeneration, leading to endplate inflammation. While MODIC changes are not directly linked to low back pain [15, 16], they have been identified as a potential risk factor for recurrent disc herniation [17].

AD’s reduction of disc space height can cause spinal instability and possibly facet loosening due to ligament laxity [18]. Consequently, this may result in segmental instability requiring additional surgical intervention or contributing to chronic pain or failed back syndrome [19]. In our study, no difference in clinical outcomes was observed between the AD and LD groups, potentially attributable to the shorter follow-up period or reduced surgical trauma. However, evidence indicates that such post-discectomy degeneration might have long-term clinical implications [20, 21]. Yorimitsu et al. [22] found that patients with over 25% loss of preoperative disc height exhibited significantly worse Japanese Orthopedic Association low back pain scores a decade post-discectomy. ​.

In both groups, long-term VAS and ODI scores remained low, aligning with findings by Soliman et al. [8]. We advocate that a genuinely minimally invasive approach to lumbar disc herniation should minimize disruption not only to soft tissue and muscle but also to the annulus and disc material.

We acknowledge that our study possesses inherent bias as it is a retrospective cohort study. Initially, we aimed to remove as much nucleus pulposus tissue as possible to prevent postoperative recurrence. Subsequently, through extensive surgical experience, we observed the herniated disc’s structure using endoscopy. In the later stages, we endeavored to preserve the annulus and the intervertebral disc’s microenvironment, a technique we categorize as limited discectomy, thus introducing some technical variations.

## Conclusion

As a conventional surgical method for treating LDH, PETD can achieve satisfactory clinical results in both LD and AD, with no significant difference in recurrence rates noted. Although AD requires a longer operation time, it does not correspondingly decrease the recurrence rate. The observed reduction in DHI and Modic changes could potentially precipitate future complications.

## Data Availability

To compare the clinical and radiological outcomes between limited discectomy (LD) and aggressive discectomy (AD) with PETD for LDH patients is ongoing. However, the dataset is available from the corresponding author upon reasonable request.

## Abbreviations

BMD: Bone mineral density
PETD: Percutaneous endoscopic transforaminal discectomy
LDH: Lumbar disc herniation
LD: Limited discectomy
AD: Aggressive discectomy
ODI: Oswestry disability index
VAS: Visual analogue scale
DHI: Disc height index
